# Immunotherapy of hepatocellular carcinoma: recent progress and new strategy

**DOI:** 10.3389/fimmu.2023.1192506

**Published:** 2023-05-10

**Authors:** Jiarui Li, Shihai Xuan, Peng Dong, Ze Xiang, Ce Gao, Mo Li, Lan Huang, Jian Wu

**Affiliations:** ^1^ Zhejiang University School of Medicine, Hangzhou, Zhejiang, China; ^2^ Department of Laboratory Medicine, The People’s Hospital of Dongtai City, Dongtai, China; ^3^ Hangzhou Institute of Cardiovascular Diseases, Affiliated Hospital of Hangzhou Normal University, Hangzhou Normal University, Hangzhou, China; ^4^ Department of Clinical Laboratory, The Affiliated Suzhou Hospital of Nanjing Medical University, Suzhou Municipal Hospital, Gusu School, Nanjing Medical University, Suzhou, Jiangsu, China

**Keywords:** hepatocellular carcinoma (HCC), immunotherapy, immune checkpoint inhibitors, adoptive cellular therapy, cancer vaccines, cytokines

## Abstract

Due to its widespread occurrence and high mortality rate, hepatocellular carcinoma (HCC) is an abhorrent kind of cancer. Immunotherapy is a hot spot in the field of cancer treatment, represented by immune checkpoint inhibitors (ICIs), which aim to improve the immune system’s ability to recognize, target and eliminate cancer cells. The composition of the HCC immune microenvironment is the result of the interaction of immunosuppressive cells, immune effector cells, cytokine environment, and tumor cell intrinsic signaling pathway, and immunotherapy with strong anti-tumor immunity has received more and more research attention due to the limited responsiveness of HCC to ICI monotherapy. There is evidence of an organic combination of radiotherapy, chemotherapy, anti-angiogenic agents and ICI catering to the unmet medical needs of HCC. Moreover, immunotherapies such as adoptive cellular therapy (ACT), cancer vaccines and cytokines also show encouraging efficacy. It can significantly improve the ability of the immune system to eradicate tumor cells. This article reviews the role of immunotherapy in HCC, hoping to improve the effect of immunotherapy and develop personalized treatment regimens.

## 1 Introduction

The liver is the sixth most common site of primary cancer in humans, and as one of the most common malignant tumors, liver cancer has the fourth most lethal rate in the world (1). It is estimated that by 2025, more than 1 million people will develop liver cancer each year (2). Among them, the incidence of hepatocellular carcinoma (HCC) accounts for 80–90% of primary liver cancer (3), with a dismal prognosis and a relative five-year survival rate of 18% (4), placing a huge load on healthcare. HCC occurs predominantly in men between the ages of 60 and 70 years (5, 6), and person with hepatitis B virus (HBV) and/or hepatitis C virus (HCV) infection, excessive alcohol use, nonalcoholic steatohepatitis (NAFLD), and a family history of liver cancer is at high risk for HCC (7–10). Although anti-HBV and anti-HCV therapy can significantly reduce the risk of HCC, it still cannot completely avoid the occurrence of HCC (11, 12). Serum alpha-fetoprotein (AFP) is a commonly used and important indicator for the diagnosis of early HCC and the monitoring of curative effect (13). However, HCC is typically detected at a level that is intermediate to advanced (14). The overall survival (OS) rate after liver resection for patients with advanced HCC has been unsatisfactory on the basis of large data (15).

**Figure 1:**
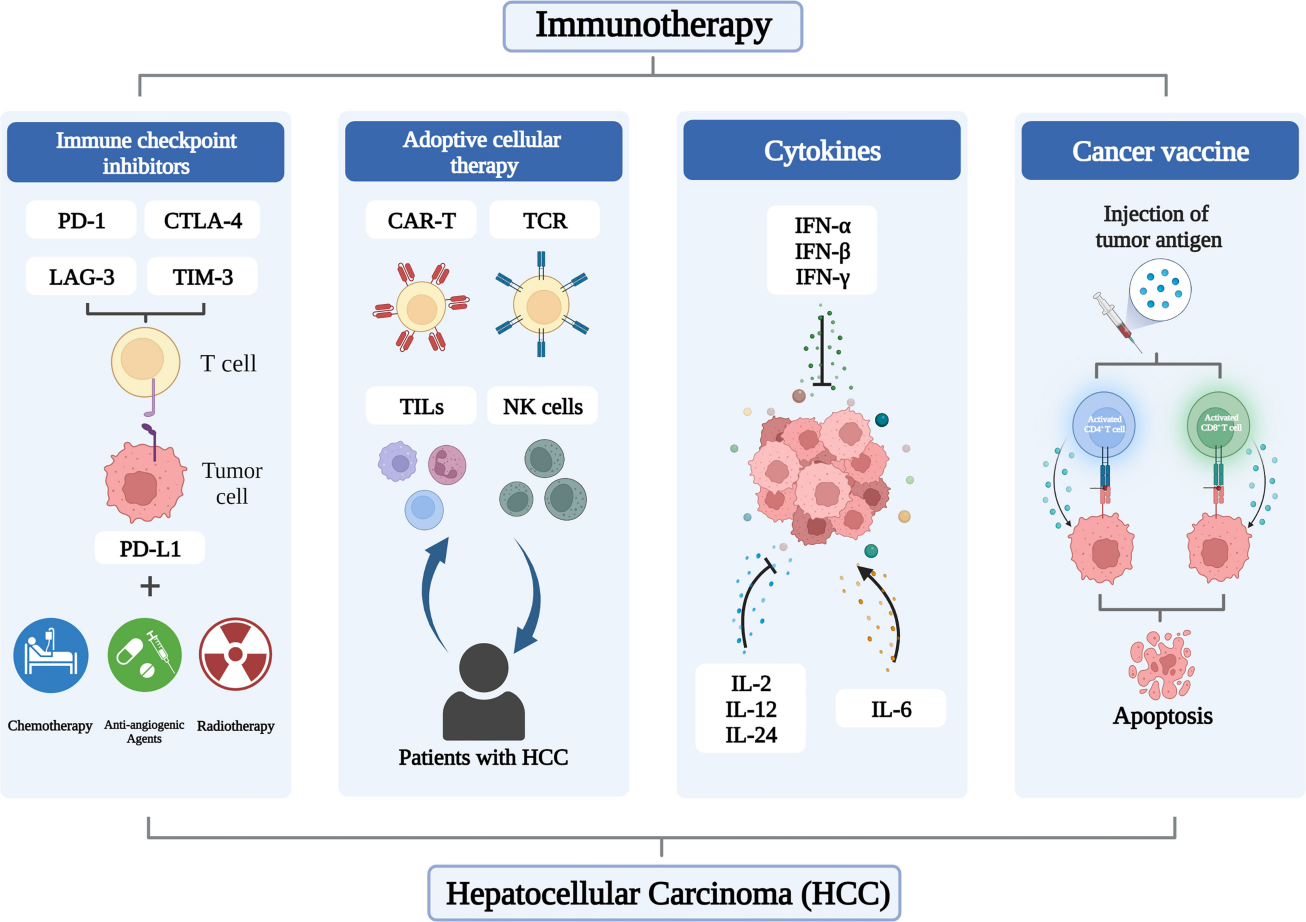
Applications of immunotherapy in HCC.

Antitumor therapies combining liver transplantation, percutaneous ablation, transarterial chemoembolization (TACE), and radiation embolization have made great strides, but the choice is largely dependent on tumor load, location, and comorbidities (16). Indeed, systemic molecular therapies have been a mainstay of treatment for advanced HCC for more than a decade, with first-line agents, including the oral multityrosine kinase inhibitors (TKIs) sorafenib, lenvatinib, and donafinib, and second-line agents, including the antiangiogenic agents regorafenib and apatinib (17). The clinical prognosis of cancer patients will be impacted by immune infiltration in the tumor microenvironment (TME), which has been proven to have a profound influence in tumor formation. An effective immune response can eradicate malignant cells or impair their phenotype and function. However, cancer cells have evolved mechanisms such as defective antigen presentation and recruitment of immunosuppressive cell populations to evade immune surveillance (18), and antitumor immune responses are suppressed. In recent years, immunotherapy, including immune checkpoint inhibitors (ICIs), adoptive cell therapy (ACT), cancer vaccines and cytokines, has shown exciting efficacy in melanoma and non-small cell lung cancer, and is revolutionizing the treatment of HCC (**Figure 1**).

This review emphasizes challenges and options to effectively treat HCC patients by describing the fundamental processes of immunotherapy and its therapeutic benefits in HCC. In addition, we suggested innovations and techniques to improve the performance of immunotherapy based on the characteristics of HCC.

## 2 Immune checkpoint inhibitors

Elimination of co-inhibitory signals is an effective way to regulate autoimmune responses. Immune checkpoint molecules are inhibitory regulatory molecules of the immune system that are critical for maintaining self-tolerance, preventing autoimmune responses, and minimizing tissue damage by controlling the timing and intensity of the immune response (19, 20). ICIs with PD-1, PD-L1, CTLA-4, TIM-3 and LAG-3 as representative targets have shown surprising safety and efficacy in the treatment of HCC (**Table 1**).

**Table 1:** Protocols to improve the efficacy of immune checkpoint inhibitors in HCC.

Target	Compounds	Model	Outcome	Reference
**PD-1**	CTLA-4	advanced HCC patients	higher ORR in the combination group a DCR of 49% & an OS rate of 40%	(21)
**PD-1**	HAIC	advanced HCC patients	better survival outcomes in the combination group	(22)
**PD-1**	HAIC	HCC patients with muscular steatosis	median PFS of 7.1 months and OS of 15.6 months	(23)
**PD-L1**	FOLFOX or GEMOX	advanced HCC patients	well tolerance of combination therapy an ORR of 26.5% and a DCR of 79.4%	(24)
**PD-1**	VEGFR-2 blockade	orthotopic HCC mouse model	vascular normalization and anti-tumor immunity promotion increased PD-L1 and PD-1 expression after VEGFR-2 blockade	(25)
**PD-1**	lenvatinib	patients with unresectable HCC	reduced expression of immune checkpoint improved cytotoxicity of T cells	(26)
**PD-1**	lenvatinib	100 patients with HCC	a PFS of 9.3 months and a median OS of 22.0 months	(27)
**PD-1**	apatinib	advanced HCC patients	enhanced efficacy in terms of ORR and OS met the primary endpoint in both the first- and second-line cohorts	(28)
**PD-1**	HAIC plus lenvatinib	advanced HCC patients	better survival outcomes in the triple therapy group a median OS of 15.9 months and median PFS of 8.8 months	(29)
**PD-1**	HAIC plus TKIs	advanced HCC patients	a median PFS of 10.6 months, ORR of 63.0% and DCR of 92.6%	(30)
**PD-1**	gallvatinib plus HAIC	treat-naive uHCC patients	the median OS and PFS were significantly higher in PLH group than that in the PL group	(31)
**PD-1**	intensive radiation therapy	patients with HCC	PD-L1 circulating tumor cells (CTCs) can be used as a predictive biomarke	(32)
**PD-1 /PD-L1**	palliative radiotherapy and antiangiogenic agents	BCLC stage c HCC	a final ORR of 40.0%, median PFS of 140 days and median OS of 637 days	(33)

TKIs, sorafenib, lenvatinib, and recorafenib; PLH, pembrolizumab plus lenvatinib with HAIC; PL, pembrolizumab plus lenvatinib without HAIC.

Inhibitors of PD-1, PDL-1, and CTLA-4 are the backbone of clinical practice or of systemic therapies in development for hepatocellular carcinoma. The CTLA-4 blocker tremelimumab’s anti-tumor and antiviral effects in HCC patients were first investigated by Bruno et al. (34). Finally, patients showed a median time to progression (TTP) of 6.48 months and a disease control rate (DCR) of 76%, revealing for the first time the transformative function of ICIs in the treatment of HCC. A phase I/II study published 4 years later by Anthony et al. first evaluated the efficacy of anti-PD-1 monoclonal antibody nabuliumab in patients with advanced HCC (35), showing an objective response rate (ORR) of 20%, a DCR of 64%, a median OS of 13.2 months as well as a median duration of response of 9.9 months, which further confirmed the potential of ICIs for HCC. In 2018, another single-arm phase II trial conducted by Colombo et al. tested the clinical efficacy and safety of pembrolizumab in 104 patients with advanced hepatocellular carcinoma who had been previously treated with sorafenib (36). The results showed an ORR rate of 17% and 44% of patients had stable disease. 77% of patients had a sustained response for ≥ 9 months, median PFS of 4.9 months, and median OS of 12.9 months, indicating that the safety and toxicity of pembrolizumab in HCC patients are manageable.

In addition to the above three types of immune checkpoints, TIM-3 and LAG-3 can also target and stimulate anti-tumor immune responses. Liu et al. (37) studied the expression, function, and regulation of the Tim-3/galectin-9 pathway in patients with HBV-related HCC, founding that the interaction of Tim-3 and galectin-9 impaired T cell effector function in HCC, and Tim-3 expression was negatively correlated with clinical outcome in patients with HBV-related HCC. LAG3 binds MHC class II molecules with high affinity, is upregulated upon T cell activation, and provides a negative signal to T cells (38). Guo et al. (39) found that serum LAG-3 levels significantly increased in HCC patients compared with healthy controls, and patients with higher LAG-3 levels had a poor prognosis after TACE. These preclinical studies demonstrated the potential of TIM-3 with LAG-3 and provided support for the subsequent combination therapy of multiple ICIs.

### 2.1 Combination of two ICIs

Single ICI may have limited efficacy in the treatment of HCC, but combinations of ICIs, such as anti-PD-1, anti-PD-L1, and anti-CTLA-4, have shown excellent efficacy in tumors such as non-small-cell lung cancer (40), melanoma (41), and colorectal cancer (42). Yau et al. (21) tested the safety of combination therapy with nivolumab (NIVO) and ipilimumab (IPI) in patients with advanced HCC and found that the ORR in the combination group was twice as high as that in the NIVO monotherapy group, with a DCR of 49% and 24-mo OS rate of 40%. This encouraging result led to accelerated FDA approval of this combination for the treatment of HCC patients after sorafenib. Single-agent pembrolizumab and nivolumab plus ipilimumab are now approved as second-line treatments for patients with disease progression from first-line TKIs (43). Liu et al. (37) found that the up-regulation of TIM-3 and/or PD-1 expression on tumor infiltrating lymphocytes (TILs) weakened their functions and was closely related to the disease progression of HBV-related HCC. The anti-tumor effect of TILs was restored after blocking TIM-3 and PD-1, which provided new ideas for the combination of immune checkpoint inhibitors. Currently, dual blockade of LAG-3 with anti-PD-1 treatment is being tested in a Phase I trial (NCT01968109) (44).

### 2.2 Combination with chemotherapy

Hepatic arterial infusion chemotherapy (HAIC) is an emerging therapy that has attracted much attention due to its high response rate and favorable survival for advanced liver cancer. In the Asian region, HAIC has been used as a sorafenib replacement therapy for patients with advanced HCC (45, 46). As a locoregional interventional therapy, HAIC not only maximizes tumor cell killing but also reduces systemic toxicity of chemotherapy agents through first-pass effects in the liver (47). Despite the promising efficacy of HAIC against unresectable tumors, its ability to inhibit extrahepatic metastasis remains unsatisfactory.

Mei et al. (22) investigated the effect of HAIC combined with anti-PD-1 immunotherapy (HAICAP) in advanced HCC and showed that patients in the HAICAP group had significantly better survival outcomes than those in the HAIC group. Muscular steatosis refers to the abnormal distribution of adipose tissue between and within muscle cells, which leads to excessive fat deposition in muscle, resulting in the decline of muscle mass, limb function and physical fitness. Yi et al. (23) evaluated the effects of anti-PD-1 immunotherapy and HAIC in HCC patients with varying degrees of muscular steatosis. The median PFS was 7.1 months and OS was 15.6 months. It was also found that patients with muscular steatosis had accelerated disease progression, increased levels of complications, prolonged hospital stay and poor prognosis after receiving treatment. A phase II study (24) focused on the clinical benefit of combining the anti-PD-L1 antibody camrelizumab with oxaliplatin-based chemotherapy (FOLFOX or GEMOX) in HCC patients and found an ORR of 26.5%, a DCR of 79.4%, a median TTR of 2.0 months, and a median PFS of 5.5 months. Camrelizumab plus FOLFOX or GEMOX chemotherapy is well tolerated and may provide a new option for patients with advanced HCC.

### 2.3 Combination with anti-angiogenic agents

HCC is a highly vascularized tumor (48), and clinical trials have successfully shown the critical role of targeting VEGF-driven angiogenesis in HCC (49). Anti-angiogenic agents inhibits tumor growth by normalizing tumor vasculature and disrupting the hypoxic tumor microenvironment (50). In 2020, atezolizumab-bevacizumab combination became the standard first-line systemic treatment for advanced HCC for its significant efficacy (43) and other combinations are being explored. Shigeta et al. (25) used orthotopic transplantation or induced mouse models of HCC to examine the effect of anti-PD-1/VEGFR-2 combination therapy on survival. VEGFR-2/PD-1 dual blockade can promote the normalization of blood vessels, reprogram the immune microenvironment, and promote the anti-tumor immunity of HCC. A nonrandomized, open-label, phase II trial by Xu et al. (28) evaluated the efficacy of camrelizumab plus apatinib in patients with advanced HCC who were naive or refractory/intolerant to first-line targeted therapy. The results showed that the combination of camrelizumab and apatinib achieved effective efficacy in terms of ORR and OS and met the primary endpoint in both the first- and second-line cohorts.

Deng et al. (26) explored the optimal combination of anti-PD-1 antibody and TKIs in patients with unresectable HCC and the possible mechanism of combined therapy. They found that lenvatinib can reduce the expression of PD-1, CTLA-4 and Tim-3 on T cells promoted by VEGFA and basic fibroblast growth factor, and improve the cytotoxicity of T cells. The combination of anti-PD-1 antibody and lenvatinib has a more effective anti-tumor effect than sorafenib or BGJ398. An Ib multicenter open-label study of 100 patients was designed to assess the tolerability, safety, and efficacy of lenvatinib plus pembrolizumab in the treatment of unresectable HCC (27). In this study, lenvatinib plus pembrolizumab produced a definitive response rate, with a PFS of 9.3 months and a median OS of 22.0 months. This suggests that lenvatinib plus PD-1 inhibition and pembrolizumab have promising antitumor effects.

### 2.4 Triple therapy

Anti-angiogenic agents combined with PD-1 or PD-L1 inhibitors have shown promising survival results in the treatment of unresectable HCC tumors. HAIC has also attracted attention for its high response rate and favorable survival in patients with advanced HCC. The combination of these three factors provides a new option for personalized treatment of different HCC patients.

A retrospective study was designed to compare survival among patients with advanced HCC who received HAIC plus lenvatinib plus a PD-1 inhibitor (HPL) with survival among patients who received lenvatinib plus a PD-1 inhibitor (PL) (29). The results showed that the survival outcome of patients in the HPL group was significantly better than that in the PL group, with a median OS of 15.9 months and a median PFS of 8.8 months, which were almost twice of PL group. All adverse events were assessed as mild and manageable, and no toxicity-related deaths occurred during follow-up. Liu et al. (30) investigated the efficacy and safety of HAIC combined with anti-PD-1 immunotherapy versus TKIs. Finally, the median PFS was 10.6 months, ORR was 63.0%, and DCR was 92.6% in the patients who received triple therapy, which was satisfying. Song et al. (31) evaluated the efficacy and safety of pembrolizumab, lenvatinib plus HAIC (PLH) versus pembrolizumab and lenvatinib (PL) in selected treat-naive uHCC patients. At the final follow-up, the median OS was 17.7 months in PLH group and 12.6 months in PL group. And the median PFS in the PLH group (10.9 months) was also significantly higher than that in the PL group (6.8 months), confirming that the combination of PD-L1 inhibitor, HAIC and lenvatinib can also improve survival rate.

### 2.5 Combination of radiotherapy

Recent studies have found that the use of local radiotherapy may stimulate antitumor immune responses by increasing apoptosis and necrosis of tumor cells and subsequently increasing the expression of antigen presentation and immunomodulatory genes (51). This may improve the response to immunotherapy, increase efficiency and reduce adverse effects. The strategy of combining immunotherapy with radiotherapy has shown promising results in clinical and basic studies. PD-L1 circulating tumor cells (CTCs) can be used as a predictive biomarker in patients with HCC who receive PD-1 inhibitors in combination with intensive radiation therapy (IMRT) and antiangiogenic therapy (32). Zhong et al. (33) explored the safety and clinical efficacy of PD-1/PD-L1 inhibitors combined with palliative radiotherapy and antiangiogenic agents in the treatment of BCLC stage C HCC. The final ORR was 40.0%, the median PFS was 140 days, and the median OS was 637 days, which proved that PD-1/PD-L1 inhibitors combined with palliative radiotherapy and anti-angiogenesis therapy were reliable without unexpected adverse events. Additional studies exploring clinical benefit are needed.

## 3 Adoptive cellular therapy

ACT is also an emerging type of immunotherapy that involves harvesting human T cells, growing them *in vitro*, increasing their number or targeted killing, and then injecting them back into the patient to kill cancer cells in the blood or tissue (52). Compared with traditional methods, ACT has the characteristics of high specificity, short onset and less interference from internal factors. Four classes of ACT that have made progress include chimeric antigen receptor T-cell (CAR-T), genetically engineered T-cell receptor (TCR), tumor-infiltrating lymphocytes (TILs) and natural killer (NK) cells. ACT has shown remarkable results in hematologic tumors, such as B-cell leukemia (53) and multiple myeloma (54).

### 3.1 Tumor-infiltrating lymphocytes and natural killer cells

TILs are lymphocytes that leave the blood and enter the tumor, and have a wide range of antigen recognition roles in tumor cells. Among them, T cell populations such as CD8 and CD4 play a key role in tumor control through mechanisms such as production of proinflammatory cytokines and promotion of plasma cell production (55, 56).

Ding et al. (57) conducted a meta-analysis of 7905 patients from 46 studies to evaluate the prognostic impact of TILs in HCC patients. The results showed that for TILs subsets, the density of CD8+, FOXP3+, CD3+ and granzyme B+ lymphocytes was significantly associated with improved survival, and the density of FOXP3+ TILs in the intratumoral (IT) was the most important prognostic marker. Higher CD8+ TIL and granzyme B+ T lymphocyte infiltration rate in the IT of patients are associated with better OS, and high CD3+ density predicts worse OS. This indicates that some TIL subsets can be used as prognostic biomarkers for HCC. Huang et al. (58) evaluated the expression of FoxP3 regulatory T cells (Tregs), CD4, CD8, and CD34 in tumor and surrounding tissues of 54 HCC patients by immunohistochemistry. It was found that the density of Tregs within the tumor was significantly elevated, whereas the density of CD8+T cells was lower. Tumor-infiltrating Tregs may promote HCC progression by promoting angiogenesis and decreasing CD8 + T cells, and are considered to be poor prognostic indicators for HCC. Whether ILs can exert normal anti-tumor activity depends on the expression level of inhibitory receptors on their surface. Pfister et al. (59) reported the progressive accumulation of depleted CD8+ PD-1 T cells in NASH-affected livers, and elimination of enriched CD8+ PD-1 T cells reduced liver injury and HCC incidence. Overall, TIL has been shown to exhibit complex anti-tumor and pro-tumor properties in HCC. As a therapeutic strategy, TILs expand immune cells from the immunosuppressive tumor microenvironment and infuse them back into the patient. However, clinical studies on the therapeutic effect of TILs in HCC are still limited.

NK cells are unique cytotoxic lymphocytes that play a crucial role in fighting tumors and infections. The importance of NK cells and their activated receptor-ligand axis in the immune surveillance of HCC has been extensively studied. Major histocompatibility complex class I chain-related protein A (MICA) is the human ligand for the NKG2D receptor on NK cells, and binding of MICA triggers NK cells and enhances antigen-specific tumor immunity (60). Reduced MICA expression in HCC tissues is associated with lower PFS and OS in patients 53 (61).

A *in vitro* study conducted by Kim et al. (62) showed that HI CD56^bright^ NK cells could produce significant killing effects on the human HCC cell line SNU398, a result that supports the next step in the investigation of the immunotherapy potential of NK cells. The clinical efficacy of immunotherapy with irreversible electroporation (IRE) in combination with allogeneic NK cells for stage IV HCC was evaluated in 40 patients (63). The results showed a synergistic effect of IRE and NK cell therapy, which not only enhanced the immune function, but also reduced the expression of AFP, showing good clinical efficacy. Weng et al. (64) randomized 85 patients with HCC after TACE and radiofrequency ablation to immunotherapy or no adjuvant therapy. Autologous cytokine-induced killer (CIK) cells were infused through the hepatic artery. CD3+, CD4+, CD56+ cells and CD4+/CD8+ ratio were increased in the CIK treatment group, while the recurrence rates at 1 year and 18 months were significantly decreased, indicating the important role of CIK in the treatment of HCC.

### 3.2 Chimeric antigen receptor T-cell and genetically engineered T-cell receptor

The major advantage of CAR-T cells, which are genetically modified lymphocytes, is the ability to recognize extracellular antigens that are presented independently of HLA without the need for antigen presentation to the surface by MHC, making more cancer cells vulnerable to attack (65). The application of CAR-T in HCC has also received extensive attention in recent years.

Secretion of IL-7 and CCL19 (7×19) improves the infiltration and survival of mouse CAR-T cells *in vivo* (66). Pang et al. (67) co-transduced T cells with CAR vector and 7×19 lentivirus to further verify the anti-HCC efficacy of 7× 19-secreting CAR-T cells. It was found that the liver tumor lesions significantly shrank on day 10 after receiving CAR-T cell injection and completely disappeared after one month. However, the number of patients enrolled in this study is small, and larger studies are still needed to prove the safety and efficacy of CAR-T cells. While HCC tumor organoids and CD39 HBV-CAR-T cells were co-cultured, Zou et al. (68) discovered that the anti-tumor effects of the CD39 HBV-CAR-T cells were enhanced when the PD-1, TIM-3, and LAG-3 checkpoints were down-regulated. Glypican-3 (GPC3) is a cell membrane cancer fetoprotein that is highly expressed in various solid tumors (69). A large body of evidence has shown that CAR-T cells targeting GPC3 can inhibit the growth of HCC cells. In a preclinical study, CAR-T cells were generated on the basis of humanized YP7 (hYP7) and HN3 antibodies (70). They found that CAR (hYP7) T cells suppressed GPC3-positive HCC in a mouse model of HCC, possibly by inducing perforin and granzyme mediated apoptosis or reducing Wnt signaling in tumor cells. Expanded and modified peripheral blood V1 T cells were employed by KAkkouk et al. (71) to produce GPC3-specific CAR and sIL-15. The generated CAR/sIL-15 V1 T cells demonstrated improved antitumor activity and successfully slowed the development of the HCC tumor. Patients with HCC may benefit from treatment with CAR-T cells that target GPC3.

The α and β chains of the TCR are key components that determine T-cell antigen specificity, and the TCR recognizes antigens through enzymatically cleaved peptides that are presented on the cell surface by MHC molecules (72). In TCR-T therapy, T cells are edited to express the TCRα and β chains, giving them tumor-targeting specificity (73).

Zhu et al. (74) identified AFP-specific TCR genes in mice and transduced human T cells with mouse TCR genes to bind HLA-A2/AFP_158_ tetamers. TCR-T cells specifically killed HLA-A2AFP HepG2 HCC tumor cells but had no obvious toxicity to normal primary hepatocytes *in vitro*. Meng et al. (75) studied the safety of HBV-TCR-T cell immunotherapy in eight patients with advanced HBV-HCC who did not qualify for liver transplantation. None of the patients showed acute adverse effects during or immediately after the injection, and they were subsequently well tolerated. The final median OS was 33.1 months, and the median TTP was 6.18 months. HBV-TCR T cells have the potential to treat HCC recurrence after liver transplantation, but their efficacy may be hindered by the immunosuppressive therapy required to prevent graft rejection. Hafezi et al. (76) engineered TCR-T cells molecularly to preserve their versatility in these patients while minimizing the risk associated with organ rejection. The results showed that patients with HBV-HCC after liver transplantation who received different immunosuppressive drugs showed different degrees of peripheral blood monocytes activation after HBV-TCR T cell infusion, and the disease progression was controlled.

## 4 Cancer vaccines

One of the key factors that needs to be addressed to achieve clinical benefit in HCC is to trigger an immune response, and cancer vaccines are an ideal immunotherapy strategy. As a key part of tumor vaccine design, tumor antigens can be traditionally divided into tumor-associated antigen (TAA) and tumor-specific antigen (TSA). There is yet little research on TSA. A preclinical experiment has demonstrated that lentinan-induced tumor-specific antigens have a significant impact on the anti-tumor immune response and immune system activation (77). Currently, the rationale for most therapeutic cancer vaccines is based on TAA to elicit an antitumor immune response to eliminate tumor cells that express these antigens (78).

A study by Mizukoshi et al. have identified some TAA in HCC and observed the activation of directed T cell responses, including Cyclophilin B, SART2, SART3, p53, MRP3, AFP and hTERT, among others (79). HepaVac-101 is a first single-arm human phase I/II multicenter cancer vaccine trial against HCC (80). 22 HCC patients were injected with the peptide antigen IMA970A and the TLR7/8/RIG I agonist CV8102, and the results showed that vaccination had a good safety profile and elicited TAA-specific immune responses in the general population of patients. Chen et al. (81) linked the XCL1 chemokine to GPC3, which is overexpressed in HCC, to construct the XCL3-GPC3 fusion molecule as a liver cancer vaccine. dendritic cells (DCS) targeted by the vaccine enhance the infiltration of antigen-specific CD8 T cells and NK cells and inhibit tumor formation and growth, with antitumor effects further enhanced by the administration of anti-PD-1. The effectiveness of cancer vaccines is limited by immunosuppressive TME, indicating the need for improvements. The effectiveness of a novel combinatorial approach based on metronomic chemotherapy and vaccination is examined in a mouse model (82). Comparing the combinatorial treatment to the vaccination alone results in a more specific T cell response, which correlates to a lower prevalence of Tregs. Such results are very encouraging and may open the door to useful advancements in immunotherapeutic approaches for HCC.

New tumor-selective vectors, key components that enhance antigen-specific immune response, can improve anticancer efficacy and circumvent systemic toxicity (83). Huang et al. (84) designed tumor-targeting lipid dendrimeric calcium phosphate (TT-LDCP) nanoparticles (NPs) using thymitine-functionalized dendrimeric polymers to efficiently deliver siRNA and pDNA into HCC cells, increase tumor invasion and CD8 T cell activation, and enhance the efficacy of cancer vaccines. And inhibit the progression of HCC. The Oxford 40 ligand (OX40L), a tumor necrosis factor receptor, is also a promising target for mRNA cancer vaccines (85). One study evaluated the anti-HCC effect and immune activation mechanism of a lipid nanoparticle-encapsulated OX40L mRNA cancer vaccine *in vitro* and *in vivo*. The results showed that the OX40L mRNA vaccine effectively induced T cell activation *in vivo* and inhibited tumor progression (86).

## 5 Cytokines

Cytokines are a class of small molecule proteins with a wide range of biological activities that are synthesized and secreted by immune cells and some non-immune cells in response to stimulation (87, 88). Cytokines mediate cell-to-cell communication and have diverse functions, including regulation of innate and adaptive immunity, cell growth, and repair of injured tissues (89). With the comprehensive development of tumor immunotherapy, cytokine therapy has entered a new era, with cytokines such as interferon (IFN) and interleukin (IL) becoming an important circuit in tumor immunotherapy (88, 90). Since HCC is characterized by a low degree of immune infiltration, the use of cytokines to expand the proliferation of immune cells and induce the recruitment of immune cells is a feasible method to improve anti-tumor immunity (**Table 2**).

**Table 2:** Different applications of cytokine in HCC.

Approach	Model	Outcome	Reference
IFN-α and sorafenib	HCC cell line Huh-7 and Sk-Hep-1	inhibited HCC cell viability, arrested cell cycle proliferation and induced cell apoptosis	(91)
pegylated IFN-α and 5-fluorouracil	HCC cell line HepG2 and BALB/c nude mice	a sharp reduction in tumor volume a significant increase in tumor cell apoptosis	(92)
IFN-β and perindopril	HCC cell line BNL.1 ME A.7R.1 and BALB/c mice	inhibited HCC development and angiogenesis suppression of vascular endothelial growth factor	(93)
IFN-γ and pentamethylquercetin	HCC cell line HepG2 and obese mice	down-regulated adipocyte induced PD-L1 expression	(94)
IL-6 blockade and sorafenib	patients with HCC	blocking of IL-6 promoted efficacy of sorafenib	(95)
IL-6 blockade and anti-PD-L1	BALB/c mice	better response to anti-PD-L1 therapy prolonged survival time of mice	(96)
IL-24	HCC cell lines and BALB/c mice	selectively infected HCC cell lines *in vitro* significantly inhibited tumor growth	(97)
IL-2	HCC cell lines and BALB/c mice	stimulated tumor-specific cytotoxic T lymphocyte responses tumor regression and long-term survival	(98)
IL-12	HCC cell lines and athymic nude mice	profound anti-proliferative and cytopathic effects on human HCC cells	(99)

### 5.1 Interferons

The discovery of IFN provided the greatest impetus for research in all cytokine studies (100). IFN can be divided into three classes: I, II and III. IFN-I is mainly expressed by innate immune cells (101), IFN-II is produced mainly by T cells and NK cells (102), whereas IFN-III expression varies according to tissue (103).

IFN-I, represented by IFN-α and IFN-β, not only affects tumor cell development through cytotoxic, cytosuppressive and antiangiogenic effects (104), but also enhances TAA expression by up-regulating MHC class I proteins (105). A frequent hallmark of noninvasive cancers is loss of IFN-I signaling. Thus, the application of IFN-I is being widely explored. Wang et al. (91) investigated the combined effects of IFN-α and sorafenib on HCC and found that the combination therapy synergistically inhibited HCC cell viability, arrested cell cycle proliferation and induced apoptosis of HCC cells by regulating the expression levels of cyclin A and cyclin B as well as pro-survival Bcl-2 family proteins. Hagiwara et al. (92) investigated the anti-HCC effect of combination therapy with pegylated interferon (PEG-IFN)-α and 5-fluorouracil (5-FU), and found that the combination group had a reduction in tumor volume and a significant increase in tumor cell apoptosis compared with the monotherapy group. This is related to the increased expression of p53 protein and mRNA induced by PEG-IFN. Noguchi et al. (93) elucidated the combined effects of the clinically used angiotensin I converting enzyme (ACE) inhibitor perindopril (PE) and IFN-β on HCC development and angiogenesis in mice. It was found that both PE and IFN-β significantly inhibited HCC occurrence and tumor neovascularization, but the effect of low-dose IFN was weaker than that of PE. Additional preclinical and clinical research is required to examine more potent combo therapies.

IFN-γ, the only member of IFN-II, also plays an important role in tumor immune regulation. Patients with HCC have been found to have fewer mucosa-associated invariant T (MAIT) cells in the peripheral blood and liver than healthy controls, and these cells produce a corresponding reduction in IFN-γ (106). Evidence suggests that interferon signaling plays a key role in regulating the efficacy and sensitivity of ICIs against a variety of tumor types. Wu et al. (107) used Kaplan-Meier survival analysis based on HCC database and found that among the nine interferon regulatory factors (IRFs) that regulate interferon signaling, decreased expression of IRF8 was associated with poor prognosis in HCC patients. However, IFN-γ and PD-1 signaling pathways were significantly inhibited in HCC patients with low IRF8, which indirectly revealed the relationship between IFN-γ and HCC prognosis. Li et al. (94) found that the natural flavonoid pentamethylquercetin (PMQ) could partially inhibit HCC progression in obese mice by down-regulating adipocyte induced PD-L1 expression through IFN-γ signaling. However, some studies have shown that IFN-γ shows a pro-tumor effect in HCC mice model. The complex role of IFN-γ in TME needs to be investigated urgently to exert its antitumor effect.

### 5.2 Interleukins

ILs are key elements for orchestrating the TME and controlling tumor-immune-cell crosstalk, enabling both a conducive environment for cancer growth and critical for an effective tumor-directed immune response (90). Therefore, these properties of ILs can be exploited to improve immunotherapy to increase effectiveness and limit side effects.

IL-6 is a multifunctional inflammatory cytokine with very low expression in normal human cells and increased concentrations in the serum of patients with hepatitis and HCC (108). The mechanism of action of IL-6 in HCC has been extensively studied. The IL-6/STAT3 signaling pathway is involved in various physiological processes, including cell growth, differentiation, and immune regulation. Aberrant activation of the IL-6/STAT3 signaling pathway has been detected in HCC (109), with an impact on the proliferation, invasion, metastasis, immune escape, and drug resistance of HCC cells (110). IL-6/STAT3 signaling upregulates HNRNPC expression in HCC cells, and knockdown of HNRNPC significantly inhibits IL-6/STAT3-enhanced HCC metastasis (111). Yang et al. (95) explored the effect of IL-6 on the sensitivity of HCC cells treated with sorafenib and its mechanism, and found that siIL-6 further promoted sorafenib to impede proliferation and induce apoptosis, suggesting that blocking IL-6 could be used as a potential therapeutic approach for sorafenib sensitivity of HCC cells. Liu et al. (96) observed that IL-6 impaired anti-tumor immunity by suppressing TNF- and IFN-expression on tumor-infiltrating CD6 cells after the co-treatment of HCC with IL-6 inhibition and anti-PD-L1 checkpoint inhibitors. Anti-PD-L1 medication had a stronger effect on tumors when paired with anti-IL-6, and mice survived noticeably longer as a result.

The possible treatment of other types of ILs for HCC has also been discussed. Deng et al. (97) constructed a recombinant oncolytic virus vaccinia based on a vaccinia virus carrying the *IL-24* gene (VG9-IL-24). Evaluation showed that VG9-IL-24 effectively infected HCC cell lines but not normal hepatocytes *in vitro*. *In vivo*, tumor growth was significantly inhibited and VG9-IL-24-treated mice lived longer. Interleukin-2 (IL-2) is an anticancer cytokine that triggers human innate and adaptive immunity by stimulating T cell proliferation and lymphocyte infiltration into tumor sites. Sun et al. (98) investigated the ability of recombinant adenovirus injection expressing IL-2 (rAd-IL-2) to inhibit tumor cell growth in HCC in HCC tumor model. The results showed that rAd-IL-2 significantly stimulated tumor-specific cytotoxic T lymphocyte responses by inducing the recruitment of CD4+ and CD8+ T cells to tumors, leading to tumor regression and long-term survival of mice during the 120-day treatment period. The study by El-Shemi et al. (99) examined the therapeutic efficacy of two armed oncolytic adenoviruses encoding the human TRAIL gene (Ad-ΔB/TRAIL) and the *IL-12* gene (Ad-ΔB/IL-12) in a preclinical model of human HCC. It was found that the combination therapy exhibited profound anti-proliferative and cytopathic effects on human HCC cells and exerted potent tumor killing effect *in vivo*. More clinical trials are still in urgent need to apply ILs to broader usage.

## 6 Conclusions

Immunotherapy is expected to achieve a new breakthrough in the radical treatment of tumors and become the mainstream method of tumor treatment because of its specificity and high efficiency, which can free the body from harmful treatment. However, due to the low degree of immune infiltration in HCC, the response to immunotherapy is limited. Although the efficacy of ICIs alone has been confirmed in dozens of clinical trials, multiple ICIs or ICIs combined with chemotherapy, anti-angiogenesis drugs, and radiotherapy are still better choices to improve ORR. In addition, immunotherapy such as ACT, tumor vaccines and cytokines are gradually being studied. However, most research has focused on the preclinical phase, and there is an urgent need to find additional therapies that are safe enough to proceed to clinical trials or to combine several classes of immunotherapies to develop personalized treatment regimens. With all the research activity in the HCC field advancing, we will undoubtedly continue to see exciting advances in immunotherapy for HCC.

## Author contributions

JL, SX and PD had the idea and drafted the work. ZX, CG and ML performed the literature search. JW and LH and critically revised the work. All authors contributed to the article and approved the submitted version.
